# A novel, non-invasive model for diagnosing liver fibrosis stage in patients with hepatocellular carcinoma

**DOI:** 10.1038/s41598-018-31351-3

**Published:** 2018-08-30

**Authors:** Gao-Xiong Ouyang, Yu-mei Zhang, Shao-Liang Zhu, Peng Wang, Yuan Ren, Jia-Hao Li, Yu-Kai Liu, Jun Chen, Bang-De Xiang, Le-Qun Li, Jian-Yong Liu, Zhi-Ming Zhang

**Affiliations:** 1 Department of Hepatobiliary Surgery, Affiliated Tumor Hospital of Guangxi Medical University, Nanning, 530021 China; 2 Department of Chemotherapy, Affiliated Tumor Hospital of Guangxi Medical University, Nanning, 530021 China; 3 Department of Radiology, Affiliated Tumor Hospital of Guangxi Medical University, Nanning, 530021 China; 4 Department of Pathology, Affiliated Tumor Hospital of Guangxi Medical University, Nanning, 530021 China

## Abstract

The aim of this study was to investigate the diagnostic value of the platelet count-to-spleen volume ratio (PSR) for diagnosing hepatic fibrosis in patients with hepatocellular carcinoma (HCC). In this interim analysis of an on-going prospective study, 117 patients with HCC and with or without cirrhosis or fibrosis in different stages were analyzed. Fibrosis staging negatively correlated with PSR and the liver volume-to-spleen volume ratio (LSR), while it positively correlated with aspartate aminotransferase-to-platelet ratio index (APRI), Frons’ index, S-index and a fibrosis index based on four factors (FIB-4). The area under the receiver operating characteristic curve (AUROC) was significantly larger for PSR (0.777) than LSR (0.633, *P* = 0.002). Among patients with significant fibrosis, AUROC for PSR did not differ significantly from the AUROCs for APRI (0.789, *P* = 0.825), Frons’ index (0.674, *P* = 0.102), FIB-4 (0.704, *P* = 0.251) or S-index (0.696, *P* = 0.204). Among patients with severe fibrosis, AUROC was significantly higher for PSR (0.808) than for LSR (0.685, *P* = 0.003), Frons’ index (0.673, *P* = 0.014), FIB-4 (0.684, *P* = 0.029), or S-index (0.672, *P* = 0.016); in contrast, the AUROC for PSR was not significantly different from that for APRI (0.739, *P* = 0.215). Among patients with cirrhosis, AUROC was significantly higher for PSR (0.814) than for LSR (0.671, *P* = 0.001) or S-index (0.679, *P* = 0.022), while the AUROC for PSR did not differ significantly from those for APRI (0.711, P = 0.105), Frons’ index (0.722, *P* = 0.061) or FIB-4 (0.708, *P* = 0.079). Our results suggest that PSR may be a useful non-invasive model for diagnosing liver fibrosis stage in patients with HCC in China.

## Introduction

Hepatocellular carcinoma (HCC) is a common malignancy and the third leading cause of cancer-related mortality worldwide; it accounts for one-fifth of all cancer cases wordwide^1^. A leading cause of HCC as well as cirrhosis is chronic hepatitis B, a disease caused by hepatitis B virus (HBV)^2^. Approximately 80% of patients with HCC in China have either cirrhosis or some degree of hepatic fibrosis. Surgical resection remains the best treatment for HCC patients who cannot undergo liver transplantation. Preoperative assessment of liver fibrosis and cirrhosis is crucial for optimizing patient prognosis. The severity of liver fibrosis and cirrhosis is closely related to preoperative hepatic reserve function as well as postoperative regeneration of remnant liver volume. Thus, this severity limits how much of the liver can be removed during hepatectomy, which can jeopardize negative margins.

The gold standard for diagnosing and staging hepatic fibrosis is liver biopsy, but in fact biopsy is rarely used because of its invasiveness and complications. The accuracy of liver biopsy is also severely compromised by intra- and inter-observer variation as well as sampling error^3–5^. This has led several studies to explore non-invasive models for diagnosing different stages of hepatic fibrosis^6–9^. These models include the aspartate transaminase-to-platelet ratio index (APRI), Frons’ index, and a fibrosis index based on four factors (FIB-4). These models are already used to evaluate liver fibrosis in patients who are chronically infected or coinfected with HBV or hepatitis C virus or who have non-alcoholic fatty liver disease (NAFLD). Indeed, the World Health Organization recommends the APRI and FIB-4 as non-invasive tools to detect significant fibrosis in resource-limited settings. To the best of our knowledge, these models have yet to be used to diagnose hepatic fibrosis in HCC patients scheduled to undergo hepatectomy.

Although APRI and FIB-4 are calculated in a straightforward way from routine blood and serum parameters, they show low sensitivity. They also appear to be inaccurate at diagnosing mild or moderate hepatic fibrosis^10,11^. Potentially more promising diagnostic indices have appeared with the advent of precise hepatectomy as a result of the increasing application of high-resolution imaging and automated image analysis^12^. For example, studies^13–15^ have indicated a correlation between hepatosplenic volume and hepatic cirrhosis. The liver-to-spleen volume ratio (LSR) has shown potential for diagnosing advanced liver fibrosis^16,17^. Another study^18^ has suggested that LSR can discriminate between advanced and severe fibrosis in patients infected with hepatitis C virus. Whether LSR can similarly diagnose the stage of hepatic fibrosis in HCC patients does not appear to have been reported.

In our efforts to improve on existing diagnostic indices of fibrosis in HCC patients, we reasoned that spleen volume and platelet count can reflect the severity of cirrhosis, and that spleen volume may be a more stable index than liver volume in HCC. Therefore we defined the platelet count-to-spleen volume ratio (PSR) as a novel, non-invasive fibrosis model and assessed its ability to diagnose liver fibrosis stage in HCC patients. We compared PSR performance with that of traditional models for diagnosing liver fibrosis stage. Our results suggest that PSR, which can be calculated more easily than other models, may be more accurate for diagnosing hepatic fibrosis stage in HCC and therefore more effective at guiding hepatectomy.

## Methods

### Ethics statement

This study was conducted in accordance with the Declaration of Helsinki and was approved by the Affiliated Tumor Hospital of Guangxi Medical University Ethnics Committee. All participants gave written informed consent for their clinical records to be used in this study.

### Patients

This report is an interim analysis of an on-going prospective study involving patients scheduled to undergo curative liver resection (defined as complete macroscopic removal of the tumor) in the Department of Hepatobiliary Surgery at the Affiliated Tumor Hospital of Guangxi Medical University. The patients in the present study were consecutively enrolled from the start of the study in October 2015 until May 2016. To be enrolled, patients had to satisfy the following inclusion criteria: (1) initial HCC diagnosis; (2) a single tumor in stage A of the Barcelona Clinic Liver Cancer (BCLC) system, which was confirmed as HCC based on postoperative pathology; (3) complete hepatic fibrosis staging based on pathology; and (4) liver computed tomography within one week before hepatectomy. Patients were excluded from the study if they (1) had another malignancy before hepatectomy; (2) received preoperative cancer treatment such as radio- or chemotherapy or transcatheter arterial chemoembolization; (3) already had undergone previous hepatectomy; or (4) had diabetes, HIV infection, or other severe disease.

### Clinical and laboratory assessment

Before hepatectomy, data on all patients were collected from medical records regarding demographics, liver biochemistry, hepatitis virus infection status, and hematological parameters. Fasting blood samples were collected between 6:30 and 8:00 a.m. on the day before hepatectomy. Liver biochemistry tests involved total bilirubin, albumin (ALB), ferritin, aspartate aminotransferase (AST), alanine transaminase (ALT), gamma-glutamyl transpeptidase (GGT), total cholesterol (TC), and prothrombin time (PT). Hematological tests assayed hemoglobin levels and determined counts of red blood cells (RBCs), white blood cell (WBCs), and platelets.

### Liver and spleen volume

**Figure 1:**
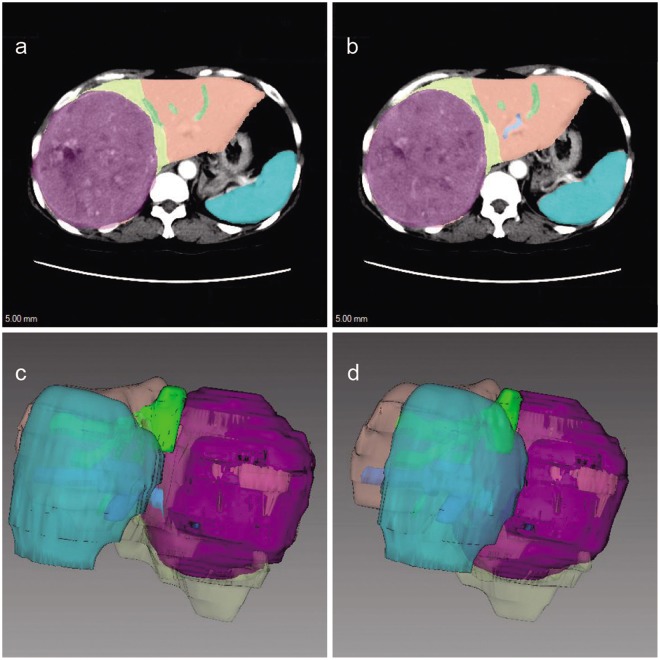
(**a**,**b**) Contours of liver and spleen filled with different colors. (**c**,**d**) Volume representing images of liver and spleen after reconstruction.

Images (5 mm thick) of the contiguous artery phase, portal venous phase and delayed phase were imported into a three-dimensional surgical simulation system (Myrian XP Liver 1.30.79.4, Intrasense, France). Portal venous phase images were adopted for image analysis and for calculation of liver and spleen volume. The contours of the liver and spleen were delineated by hand using a computer mouse, the areas within the contours of liver and spleen were filled with different colors, and the gallbladder and inferior vena cava were eliminated from each slice. The volumes enclosed by the liver and spleen contours were automatically calculated using the Myrian XP Liver software (Fig. 1).

### Histological assessment

Specimens of resected liver tissue were fixed in 10% buffered formalin, embedded in paraffin, and stained with hematoxylin and eosin (HE), Masson’s trichrome and reticular fiber staining. Diagnosis was confirmed based on analysis of at least 1.5 cm of liver tissue containing at least five portal tracts. Two histologists independently determined the extent of hepatic fibrosis in each sample using the METAVIR scoring system^19^: F0, no fibrosis; F1, expansion of portal zones; F2, expansion of most portal zones and occasional bridging; F3, expansion of most portal zones and marked bridging and occasional modules; F4, cirrhosis. Histologists were blinded to patients’ results for hepatic fibrosis models. Disagreements between the histologists were resolved by consensus.

### Hepatic fibrosis models

Patients were assessed using the following hepatic fibrosis models^7,20–22^:

$$\begin{array}{rcl}{\rm{PSR}} & = & {\rm{platelet}}\,{\rm{count}}\,({10}^{9}/{\rm{L}})/{\rm{spleen}}\,{\rm{volume}}\,(\mathrm{ml})\\ {\rm{LSR}} & = & {\rm{liver}}\,{\rm{volume}}\,({\rm{ml}})/{\rm{spleen}}\,{\rm{volume}}\,({\rm{ml}})\\ {\rm{APRI}} & = & 100\times ({\rm{AST}}\,({\rm{IU}}/{\rm{L}})/{\rm{upper}}\,{\rm{normal}}\,{\rm{limit}}\,{\rm{of}}\,{\rm{AST}})\\  &  & /{\rm{platelet}}\,{\rm{count}}\,({10}^{9}/{\rm{L}})\\ {\rm{Frons}}\mbox{'}\,{\rm{index}} & = & 7.811-3.131\times {\rm{In}}\,(\mathrm{platelet}\,{\rm{count}}\,({10}^{9}/{\rm{L}}))+0.781\\  &  & \times \,{\rm{In}}\,({\rm{GGT}}\,(\mathrm{IU}/{\rm{L}}))+3.467\times {\rm{In}}\,(\mathrm{age}\,({\rm{year}}))-0.014\times ({\rm{TC}}).\\ {\rm{FIB}}-4 & = & ({\rm{age}}\,(\mathrm{year})\times {\rm{AST}}\,({\rm{IU}}/{\rm{L}}))/({\rm{platelet}}\,{\rm{count}}\,({10}^{9}/{\rm{L}})\\  &  & \times \,{({\rm{ALT}}(\mathrm{IU}/{\rm{L}}))}^{1/2})\\ {\rm{S}}-{\rm{index}} & = & 1000\times {\rm{GGT}}\,({\rm{IU}}/{\rm{L}})/({\rm{PLT}}\,({10}^{9}/{\rm{L}})\times {({\rm{ALB}}({\rm{g}}/{\rm{L}}))}^{2})\end{array}$$

### Statistical analysis

Continuous variables were expressed as mean ± standard deviation, while categorical variables were expressed as number (percentage). Univariate analysis was performed using one-way ANOVA or, in the case of continuous variables showing a skewed distribution, the nonparametric Kruskal-Wallis H test in order to identify significant differences in the ability of the six hepatic fibrosis models (PSR, LSR, APRI, Frons’ index, FIB-4, S-index) to differentiate patients in different stages of liver fibrosis. Correlations between hepatic fibrosis models and liver fibrosis stage were assessed using the Spearman rank correlation coefficient. These statistical analyses were performed using SPSS 23.0.

Diagnostic accuracy of each hepatic fibrosis model was assessed based on the area underneath the receiver operating characteristic curve (AUROC). The sensitivity, specificity, positive predictive value, negative predictive value and AUROC were determined for each fibrosis model in the case of patients with significant fibrosis (defined as ≥F2), severe fibrosis (≥F3) or cirrhosis (F4). Receiver operating characteristic curves were analyzed statistically using Medcalc 15.2.0.

All P values are 2-sided, and P < 0.05 was considered statistically significant.

## Results

**Figure 2:**
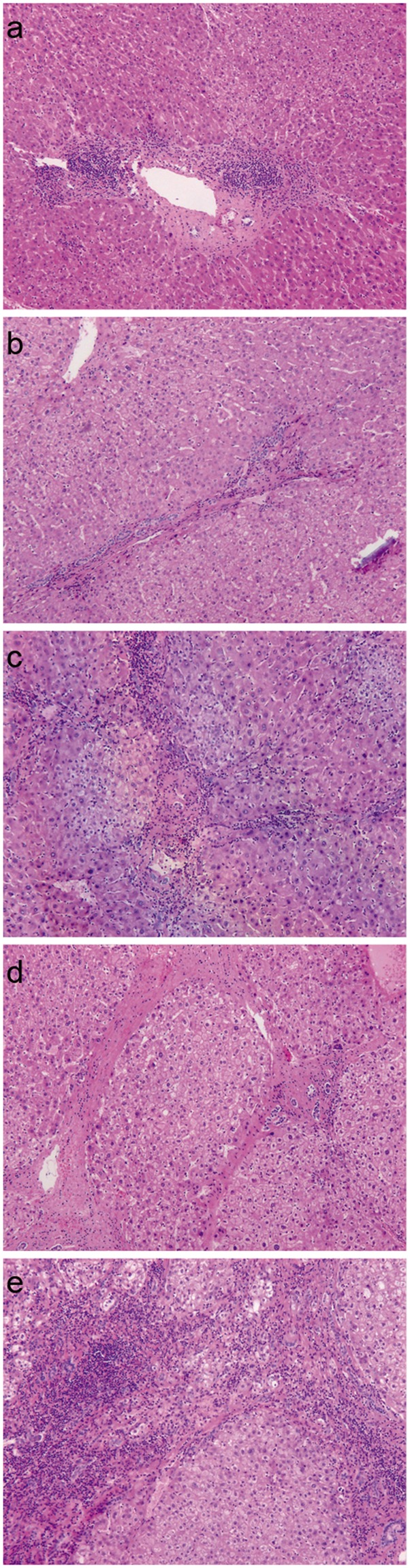
Postoperative pathological hepatic fibrosis staging. (**a**) Fibrosis stage F0 (HE × 40); (**b**) Fibrosis stage F1 (HE × 40); (**c**) Fibrosis stage F3 (HE × 40); (**d**) Fibrosis stage F4 (HE × 40); (**e**) Fibrosis stage F5 (HE × 40)).

Table 1 shows the main demographic and clinical characteristics of the study population of 117 patients, including 98 (83.76%) men and 19 (16.24%) women. Of all 117 patients, 96 (82.05%) were HBsAg-positive and 110 (94.02%) had Child-Pugh class A liver function, while the remaining 7 (5.98%) had Child-Pugh class B liver function. The distribution of METAVIR scores for hepatic fibrosis was as follows: F0, 6 (5.13%) patients; F1, 29 (24.79%) patients; F2, 25 (21.37%) patients; F3, 21 (17.95%) patients; and F4, 36 (30.77%) patients. Figure 2 shows representative pathology images from patients with the different METAVIR scores. Given the small number of F0 patients, we pooled F0 and F1 patients into a group with “no significant fibrosis” (F0-F1).

**Table 1:** The characteristic of patients in this study.

Variables	n
Gender	
Male (n, %)	98 (83.76)
Female (n, %)	19 (16.24)
Age (year)	50.50 ± 10.31
Weight (KG)	62.86 ± 9.94
Height (CM)	164.68 ± 6.51
WBC count (10^9^/L)	6.71 ± 2.42
RBC count (10^12^/L)	4.77 ± 0.65
Hemoglobin (g/L)	138.13 ± 18.28
PLT (10^9^/L)	224.75 ± 95.01
Total bilirubin (umol/L)	16.18 ± 30.21
Albumin (g/L)	39.08 ± 4.21
Ferritin (ug/L)	382.21 ± 207.58
ALT (IU/L)	41.62 ± 39.51
AST (IU/L)	44.91 ± 39.29
GGT (IU/L)	88.80 ± 130.40
TC (mmol/L)	1.76 ± 6.85
PT (s)	12.98 ± 1.30
HBsAg (+) (n, %)	96 (82.05)
HBsAg (−) (n,%)	21 (17.95)
Child-Pugh Classification	
A (n,%)	110 (94.02)
B (n,%)	7 (5.98)
Tumor size (cm)	5.35 ± 2.28
Liver volume (ml)	1186.92 ± 262.01
Spleen volume (ml)	219.54 ± 111.69
Hepatic fibrosis stage	
F0	6 (5.13)
F1	29 (24.79)
F2	25 (21.37)
F3	21 (17.95)
F4	36 (30.77)
PSR	1.42 ± 0.95
LSR	6.52 ± 3.08
APRI	0.66 ± 0.49
Frons’index	7.45 ± 1.65
FIB-4 index	2.03 ± 1.31
S-index	0.31 ± 0.33

**Figure 3:**
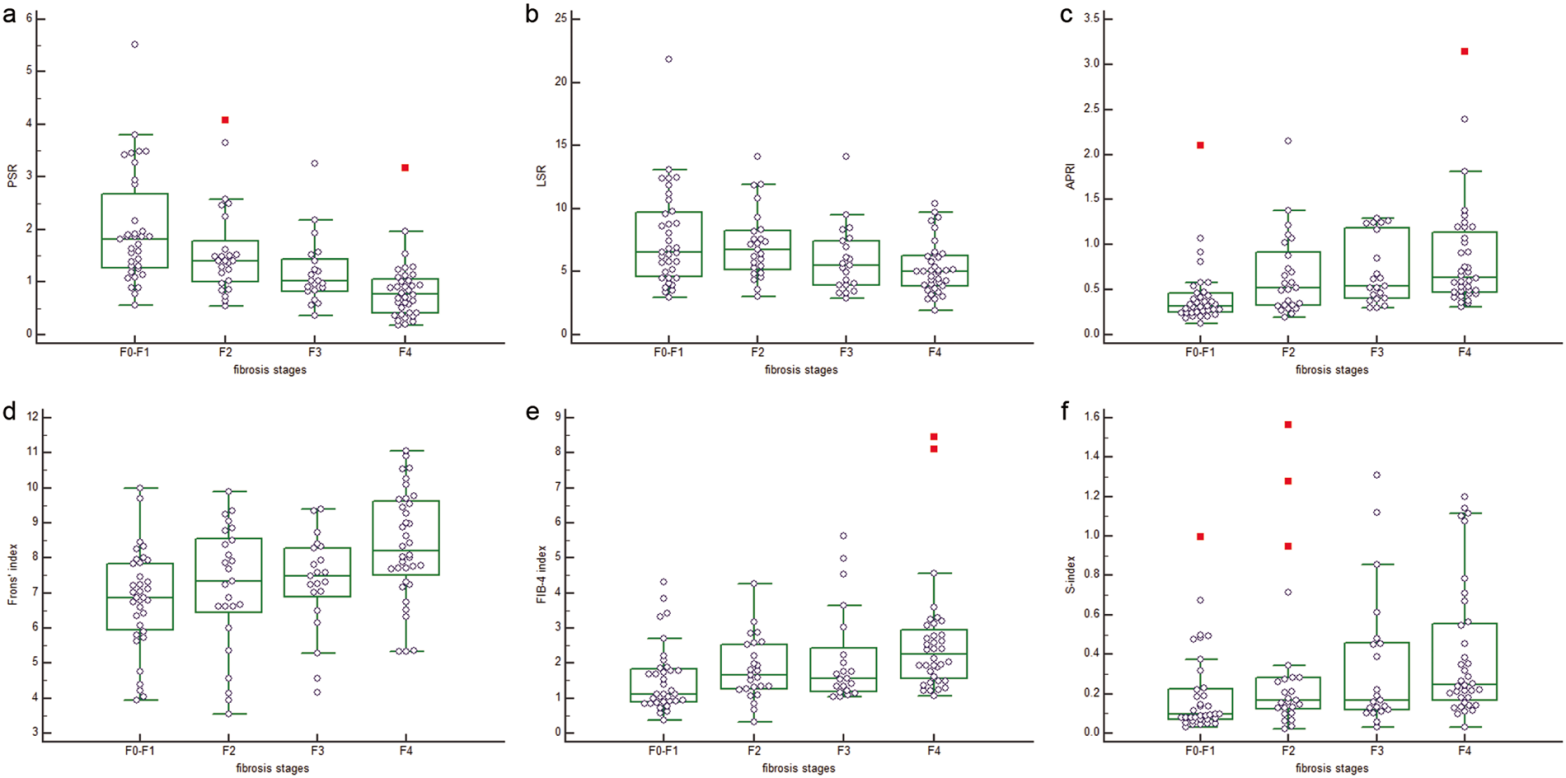
Box plots of the comparison of liver fibrosis models scores of different liver stages. (**a**) PSR, *P* < 0.001; (**b**) LSR, *P* = 0.006; (**c**) APRI, *P* = 0.002; (**d**) Frons’ index, *P* = 0.001; (**e**) FIB-4 index, *P* = 0.001; and (**f**) S-index, *P* = 0.002).

Potential correlation between liver fibrosis models and fibrosis stage based on postoperative pathology was explored using Spearman rank correlation (Table 2). Fibrosis staging negatively correlated with PSR (r = −0.577) and LSR (r = −0.312), while it positively correlated with APRI (r = 0.476), Frons’ index (r = 0.366), FIB-4 (r = 0.384) and S-index (r = 0.353). PSR differed significantly among the four subgroups of patients with no significant fibrosis, significant fibrosis, severe fibrosis or cirrhosis, and it differed significantly between all pairs of these four subgroups. The other indices tested in this study (LSR, APRI, Frons’ index, FIB-4, S-index) did not differ significantly from PSR for any of the four patient subgroups (P < 0.05, Table 3 and Fig. 3).

**Table 2:** Correlation of liver fibrosis models scores with METAVIR scores.

Variables	spearman r	(95% CI)	*P*-value
PSR	−0.577	−0.687–0.442	<0.05
LSR	−0.312	−0.467–0.139	<0.05
APRI	0.476	0.322–0.605	<0.05
Frons’ index	0.366	0.198–0.513	<0.05
FIB-4 index	0.384	0.217–0.528	<0.05
S-index	0.353	0.183–0.502	<0.05

**Table 3:** Liver fibrosis models scores of different liver stages.

Variables	F0-F1	F2	F3	F4	F/X2-value	*P*-value
PSR	2.008 ± 1.078	1.603 ± 0.880	1.195 ± 0.648	0.842 ± 0.567	12.60	<0.05
LSR	7.681 ± 3.876	7.137 ± 2.737	5.956 ± 2.679	5.291 ± 2.070	12.54	<0.05
APRI	0.428 ± 0.357	0.661 ± 0.457	0.704 ± 0.372	0.857 ± 0.598	5.09	<0.05
Frons’ index	6.816 ± 1.444	7.165 ± 1.784	7.304 ± 1.381	8.363 ± 1.547	16.74	<0.05
FIB-4 index	1.547 ± 0.956	1.834 ± 0.890	2.150 ± 1.386	2.578 ± 1.612	17.49	<0.05
S-index	0.200 ± 0.216	0.318 ± 0.392	0.349 ± 0.358	0.403 ± 0.344	14.65	<0.05

**Figure 4:**
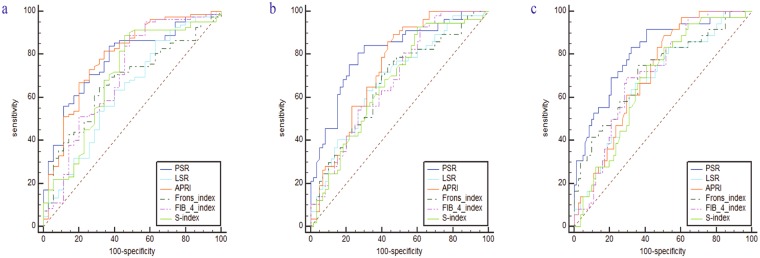
Receiver operating characteristic curves of hepatic fibrosis models for evaluating significant fibrosis (**a**) severe fibrosis (**b**) and cirrhosis (**c**).

Next we compared the models for their ability to diagnose significant fibrosis, severe fibrosis, and cirrhosis (Table 4 and Fig. 4). AUROC was significantly higher for PSR than for LSR among patients with significant fibrosis (0.777 vs. 0.633, Z = 3.096, *P* = 0.002), severe fibrosis (0.808 vs. 0.685, Z = 2.992, *P* = 0.003), or cirrhosis (0.814 vs. 0.671, Z = 3.511, *P* < 0.001). Among patients with significant fibrosis, AUROC for PSR (0.777) did not differ significantly from AUROCs for APRI (0.789, Z = 0.221, *P* > 0.05), Frons’ index (0.674, Z = 1.636, *P* > 0.05), FIB-4 (0.704, Z = 1.147, *P* > 0.05), or S-index (0.696, Z = 1.269, *P* > 0.05). Among patients with severe fibrosis, AUROC for PSR (0.808) was not significantly different from that for APRI (0.739, Z = 1.24, *P* > 0.05), but it was significantly higher than that for Frons’ index (0.673, Z = 2.468, *P* < 0.05), FIB-4 (0.684, Z = 2.191, *P* < 0.05), and S-index (0.672, Z = 2.401, *P* < 0.05). Among patients with cirrhosis, AUROC for PSR (0.814) was not significantly different from that for APRI (0.711, Z = 1.623, *P* > 0.05), Frons’ index (0.722, Z = 1.876, *P* > 0.05), or FIB-4 (0.708, Z = 1.756, *P* > 0.05), but it was significantly higher than that for S-index (0.679, Z = 2.286, *P* < 0.05).

**Table 4:** The diagnostic value of hepatic fibrosis models for evaluating significant fibrosis, severe fibrosis and cirrhosis.

Fibrosis stage	Variables	Cut-off	AUROC (95% CI)	Se (%)	Sp (%)	PPV (%)	NPV (%)	*P-*value
≥F2	PSR	1.531	0.777 (0.691–0.849)	84.15	62.86	69.38	79.86	<0.05
	LSR	8.459	0.633 (0.539–0.720)	86.59	37.14	57.94	73.47	<0.05
	APRI	0.437	0.789 (0.704–0.859)	73.17	74.29	74.00	73.47	<0.05
	Frons’ index	7.317	0.674 (0.581–0.757)	64.63	68.57	67.28	65.97	<0.05
	FIB-4 index	1.121	0.704 (0.612–0.785)	89.02	51.43	64.70	82.41	<0.05
	S-index	0.099	0.696 (0.605–0.778)	89.02	54.29	66.07	83.18	<0.05
≥F3	PSR	1.29	0.808 (0.725–0.875)	84.21	70.00	73.73	81.59	<0.05
	LSR	6.169	0.685 (0.593–0.768)	71.93	60.00	64.26	68.13	<0.05
	APRI	0.365	0.739 (0.649–0.815)	89.47	53.33	65.72	83.51	<0.05
	Frons’ index	7.234	0.673 (0.581–0.757)	75.44	56.67	63.52	69.76	<0.05
	FIB-4 index	1.121	0.684 (0.592–0.767)	92.98	38.33	60.12	84.52	<0.05
	S-index	0.099	0.672 (0.579–0.756)	92.98	40.00	60.78	85.07	<0.05
F4	PSR	1.138	0.814 (0.731–0.880)	83.33	67.90	72.19	80.29	<0.05
	LSR	5.377	0.671 (0.578–0.755)	66.67	65.43	65.85	66.25	<0.05
	APRI	0.402	0.711 (0.620–0.791)	88.89	49.38	63.72	81.63	<0.05
	Frons’ index	7.675	0.722 (0.632–0.801)	75.00	64.20	67.69	71.97	<0.05
	FIB-4 index	1.872	0.708 (0.617–0.789)	69.44	70.37	70.09	69.72	<0.05
	S-index	0.175	0.679 (0.587–0.762)	75.00	59.26	64.80	70.33	<0.05

Among patients with significant fibrosis, AUROC for LSR (0.633) was significantly lower than that for APRI (0.789, Z = 2.465, *P* = 0.014), but it was not significantly different from that for Frons’ index (0.674, Z = 0.527, *P* > 0.05), FIB-4 (0.704, Z = 0.355, *P* > 0.05), or S-index (0.696, Z = 0.792, *P* > 0.05). Among patients with severe fibrosis, AUROC for LSR (0.685) was not significantly different from that for APRI (0.739, Z = 0.843, *P* > 0.05), Frons’ index (0.673, Z = 0.175, *P* > 0.05), FIB-4 (0.684, Z = 0.013, *P* > 0.05) or S-index (0.672, Z = 0.183, *P* > 0.05). Similarly, among patients with cirrhosis, AUROC for LSR (0.671) was not significantly different from that for APRI (0.711, Z = 0.578, *P* > 0.05), Fron’s index (0.722, Z = 0.749, *P* > 0.05), FIB-4 (0.708, Z = 0.532, *P* > 0.05), or S-index (0.679, Z = 0.118, *P* > 0.05).

## Discussion

Hepatectomy remains the first choice for treating HCC in patients who do not undergo liver transplantation, although radio-, chemo-, bio-, and immunotherapy are also widely used^23^. The efficacy of these various approaches is still unsatisfactory. The main cause of perioperative death is postoperative liver failure, which is associated with degree of hepatitis or cirrhosis in many HCC patients in China. The severity of hepatitis and cirrhosis affects preoperative liver functional reserve, the liver volume that can be safely removed during hepatectomy, and postoperative residual liver regeneration. This highlights the importance of preoperative assessment of hepatic fibrosis stage in HCC patients.

Liver biopsy remains the main method for diagnosing hepatic fibrosis stage, but its invasiveness and associated complications make it less widely used. As non-invasive alternatives, LSR and APRI and other indices have been tested for their ability to evaluate hepatic fibrosis in patients with chronic liver disease. Here we provide one of the few reports assessing the ability of these indices to diagnose hepatic fibrosis in HCC patients scheduled to undergo hepatectomy. Furthermore, we describe the novel model PSR and provide evidence that it may perform even better than LSR and other indices for patients with different extents of hepatic fibrosis. The results may help guide rational and scientific surgical planning and postoperative treatment.

In our study, fibrosis stage showed a significant negative correlation with PSR and LSR, but a significant positive correlation with APRI, Frons’ index, FIB-4, and S-index. While these results cannot be directly compared with other studies, since those studies did not examine patients with HCC, the data are consistent with previous work showing an association between these indices and fibrosis stage in other types of patients. One study^18^ reported a significant negative correlation between LSR and hepatic fibrosis stage in patients infected with hepatitis C virus. Another study^17^ reported a strong correlation between LSR and fibrosis stage in patients who were suspected of having chronic liver disease or who had focal hepatic lesions. Other work^8,24^ reported a correlation of APRI and FIB-4 with fibrosis stage in HBeAg-negative patients with chronic hepatitis B and ALT ≤2 (upper normal limit) as well as in patients with chronic hepatitis B and non-alcoholic fatty liver disease. In this way, our results as well as the literature indicate that the fibrosis models in our study may be used to diagnose hepatic fibrosis stage in HCC patients.

The finding in our studies and others’ work that LSR and PSR gradually diminish with hepatic fibrosis progression may reflect reduced blood perfusion in the portal vein and hepatic venous out-flow^25^. Reduction in the branches of the intrahepatic portal vein and in the hepatic vascular bed as well as expansion of the fiber matrix lead to portal hypertension and liver atrophy. Portal hypertension then leads to spleen hypertrophy, further reducing platelet count^26,27^.

PSR relies on routine serum and biochemical parameters and on straightforward measurement of liver and spleen volume using widely available 3D reconstruction technology and 3D surgery simulation systems. On the other hand, the time-consuming posting requested when using 3D simulation volumetry to calculate spleen and liver volumes may be a barrier to potential widespread use.

Our finding that PSR can be used to diagnose hepatic fibrosis stage in patients with HCC is consistent with studies showing that spleen volume and platelet count are closely related to hepatic fibrosis. When we compared PSR with other hepatic fibrosis models, we obtained different results depending in the extent of fibrosis. Among patients with significant fibrosis, AUROC for PSR was not significantly different from that for APRI, Frons’ index, FIB-4, or S-index. Among patients with severe fibrosis, AUROC for PSR was not significantly different from that for APRI, but it was significantly higher than that for Frons’ index, FIB-4 and S-index. Among patients with cirrhosis, AUROC for PSR was not significantly different from that of APRI, Frons’ index, or FIB-4, but it was significantly higher than that for S-index.

These results suggest that PSR may be more stable than APRI, Frons’ index, FIB-4, and S-index for evaluating liver fibrosis and cirrhosis in HCC patients. In addition, PSR appears to show greater diagnostic value than LSR for diagnosing significant fibrosis, severe fibrosis, or cirrhosis. This may be because LSR involves total liver volume, which is closely related to tumor size in HCC patients. In addition, HCC cells may secrete active substances to promote the proliferation of normal liver tissue, which may help to explain why liver volume is less stable than spleen volume. The other indices tested in this study (APRI, Frons’ index, FIB-4, S-index) may be less reliable than PSR because they rely on multiple serum and biochemical parameters (e.g. AST, ALT, GGT), which may vary with many factors such as blood drawing time, sample delivery time, cancer treatment, tumor size, and liver functional status.

We emphasize that the results reported here are interim findings in an on-going prospective trial that will ultimately analyze a larger sample. Conclusions therefore depend on subsequent validation with a larger study population. Given the interim nature of this analysis, we chose not to correct for potential confounding due to factors such as sex, age, or body mass index. In any event, it seems less likely that these factors affect our results. For example, we are unaware of evidence linking spleen volume to age, sex or BMI, and studies^17,18^ evaluating LSR and hepatic fibrosis in patients with chronic hepatitis B do not adjust for these factors. Similarly, the World Health Organization recommendations for using APRI to detect significant fibrosis in resource-limited settings do not involve correcting for these three factors. Nevertheless, such confounding analysis should be performed with a larger sample.

In addition to our relatively small sample, our analysis is limited by the fact that we included only patients with single tumors in BCLC stage A. Our results should be verified in patients with multiple tumors. It will also be interesting to see whether PSR can evaluate hepatic fibrosis stage in patients in whom the portal vein is compressed by a large tumor, causing portal hypertension that ultimately leads to spleen hypertrophy. Such work will be important for establishing PSR as an easily calculated, non-invasive model for diagnosing hepatic fibrosis stage in HCC patients in China.
